# Borealin/CDCA8 deficiency alters thyroid development and results in papillary tumor-like structures

**DOI:** 10.3389/fendo.2023.1286747

**Published:** 2023-10-27

**Authors:** Hortense Didier-Mathon, Athanasia Stoupa, Dulanjalee Kariyawasam, Sonny Yde, Beatrix Cochant-Priollet, Lionel Groussin, Frédéric Sébag, Nicolas Cagnard, Patrick Nitschke, Dominique Luton, Michel Polak, Aurore Carré

**Affiliations:** ^1^ Université Paris Cité, Centre National de la Recherche Scientifique (CNRS), Institut National de la Santé et de la Recherche Médicale (INSERM), Institut Cochin, Paris, France; ^2^ IMAGINE Institute Affiliate, Paris, France; ^3^ Pediatric Endocrinology, Gynecology and Diabetology Department, Hôpital Universitaire Necker-Enfants Malades, Assistance Publique Hopitaux de Paris (AP-HP), Paris, France; ^4^ Université Paris Cité, Faculté de Médecine, Paris, France; ^5^ Department of Pathology, Cochin Hospital, Assistance Publique Hopitaux de Paris (AP-HP) Centre, Paris, France; ^6^ Department of Endocrinology, Université Paris Cité, Cochin Hospital, Assistance Publique Hopitaux de Paris (AP-HP) Centre, Paris, France; ^7^ Endocrine Surgery, Conception University Hospital, Aix-Marseille University, Marseille, France; ^8^ Bioinformatics Platform, Institut Imagine, Institut National de la Santé et de la Recherche Médicale (INSERM) UMR 1163, Paris, France; ^9^ Département de Gynécologie Obstétrique, Hôpital Bicêtre, Assistance Publique Hopitaux de Paris (AP-HP) Le Kremlin Bicêtre France, Université Paris Saclay, Le Kremlin Bicêtre, France; ^10^ Centre de référence des maladies endocriniennes rares de la croissance et du développement, Necker-Enfants Malades University Hospital, Paris, France; ^11^ Centre régional de dépistage néonatal (CRDN) Ile de France, Paris, France

**Keywords:** Borealin, congenital hypothyroidism, thyroid cancer, thyroid dysgenesis, thyroid function

## Abstract

**Background:**

*BOREALIN*/*CDCA8* mutations are associated with congenital hypothyroidism and thyroid dysgenesis. Borealin is involved in mitosis as part of the Chromosomal Passenger Complex. Although *BOREALIN* mutations decrease thyrocyte adhesion and migration, little is known about the specific role of Borealin in the thyroid.

**Methods:**

We characterized thyroid development and function in Borealin-deficient (*Borealin* ^+/−^) mice using histology, transcriptomic analysis, and quantitative PCR.

**Results:**

Thyroid development was impaired with a hyperplastic anlage on embryonic day E9.5 followed by thyroid hypoplasia from E11.5 onward. Adult *Borealin* ^+/−^ mice exhibited euthyroid goiter and defect in thyroid hormone synthesis. *Borealin* ^+/−^ aged mice had disorganized follicles and papillary-like structures in thyroids due to ERK pathway activation and a strong increase of *Braf*-like genes described by The Cancer Genome Atlas (TCGA) network of papillary thyroid carcinoma. Moreover, *Borealin* ^+/−^ thyroids exhibited structural and transcriptomic similarities with papillary thyroid carcinoma tissue from a human patient harboring a *BOREALIN* mutation, suggesting a role in thyroid tumor susceptibility.

**Conclusion:**

These findings demonstrate Borealin involvement in critical steps of thyroid structural development and function throughout life. They support a role for Borealin in thyroid dysgenesis with congenital hypothyroidism. Close monitoring for thyroid cancer seems warranted in patients carrying *BOREALIN* mutations.

## Introduction

Congenital hypothyroidism (CH) is among the most common preventable causes of intellectual disability, reflecting the critical role of the thyroid hormones thyroxine (T4) and triiodothyronine (T3) in brain development. In France, where routine neonatal screening was started in 1978, CH is found in 1/3,000 neonates, with an increase in recent decades (1). Thyroid dysgenesis (TD) accounts for approximately 65% of CH cases and hormone-synthesis abnormalities for the remaining 35%. TD may be initiated at any step during thyroid development and, therefore, has widely variable manifestations including athyreosis (21%), ectopic thyroid (41%), and thyroid hypoplasia or hemithyroid (3%) (2, 3). In humans, the thyroid anlage on the midline starts to invaginate from the floor of the foregut at about embryonic day (E) 22 and expresses NKX2-1, PAX8, and FOXE1 from E32–33 (E8.5–9.5 in mice) onward (4, 5). Starting on E26, the ultimobranchial bodies (lateral anlage) develop from the fourth pharyngeal pouch on each side (6). The midline and lateral anlages migrate actively and then fuse in the definitive pretracheal position on E44 (E13.5 in mice) (7). The cells differentiate into thyrocytes, which produce thyroglobulin (TG) and T4 starting at 8 and 11 gestational weeks, respectively, (E16.5 for T4 production in mice) (8). Thyrocytes also express thyroid peroxidase (TPO), which contributes to T4 synthesis, and the sodium/iodide symporter NIS, with iodide being the limiting factor in T4 synthesis.

Thyroid cancers, except medullary carcinoma, are derived from thyrocytes, and 80% are papillary thyroid carcinoma (PTC) (9). Genetic studies report high frequency (70%) of somatic alterations of genes coding for effectors in the mitogen-activated protein kinase (MAPK) signaling pathway, including point mutations of *BRAF*. BRAF is a serine- or threonine-specific protein kinase activated by RAS, which results in a phosphorylation cascade leading to MAPK activation, which regulates cell division and survival. All *BRAF* genetic alterations lead to constitutive activation of the kinase with stimulation of the MAPK pathway including ERK phosphorylation and cause oncogenic transformation. BRAF^V600E^ is the most frequent genetic alteration found in PTC (40%–60%) (10). ERK is negatively feedback-regulated by Dual Specificity Phosphatases (DUSPs), especially two ERK-specific DUSPs, DUSP5 and DUSP6. DUSP5 and DUSP6 mRNA levels are markers of activation of the MAPK signaling pathway in PTC (11).

We have reported three missense *BOREALIN/CDCA8* mutations in patients with CH and TD (c.443C>T, p.S148F; c.341G>A, p.R114Q; and c.530T> G, p.L177W) (12). Another *BOREALIN* mutation, a splice site mutation:c.585-1G>C in intron 7, was also identified in a patient with CH and TD (13). Borealin/CDCA8 is a component of the Chromosomal Passenger Complex (CPC), which is involved in various steps of mitosis and makes a major contribution to cytokinesis coordination (14, 15),. We have demonstrated that Borealin is also involved in thyrocyte adhesion and migration (12).

*BOREALIN* mutations are mono- or biallelic and have incomplete penetrance, thus producing TD with or without CH (12). *In vitro* studies of functional effects found no differences in severity across *BOREALIN* mutations and lead to loss of function of BOREALIN with decrease of thyrocyte adhesion and migration (12). Accordingly, homozygous and heterozygous mutations produce similar phenotypes in humans. In addition, a link to carcinogenesis is suggested by a report of the mother of a CHTD patient harboring a heterozygous mutation, c.341G>A, p.R114Q, having asymmetric thyroid lobes and who has developed PTC later during adulthood (12).

The objective of this experimental study was to shed light on the role of Borealin in thyroid development and function. We used mice deficient in Borealin. Homozygous Borealin knockout mice die during early embryogenesis, on E5.5, in keeping with the crucial role of Borealin in development (16). We therefore studied heterozygous *Borealin* ^+/−^ mice throughout intrauterine development and postnatal life. As patients carried heterozygous *BOREALIN* mutation, analyzing *Borealin* ^+/−^ mice was legitimate. We also investigated thyroid samples from a patient with a heterozygous c.341G>A, p.R114Q mutation, and papillary thyroid carcinoma (12). Our findings support a role for Borealin in thyroid development, function, and abnormal architecture.

## Material and methods

### Animals

Borealin-deficient mice generated as previously described were donated by the RIKEN Center for Genomic Medicine (Yokohama, Kanagawa 230-0045, Japan) (16). Briefly, a lacZ cDNA reading frame and a neomycin resistance cassette were inserted into the first exon of *Borealin/Cdca8* to disrupt the ATG codon and null allele of the *Borealin* gene.

Experiments using mice were certified by the Direction Departementale de la Protection des Populations for the French Ministry of Research, Health and Agriculture (Paris) under agreement number A75-13–19 in accordance with approved guidelines of French and European legislation. The animals were housed in a temperature-controlled room on a 12:12-h light–dark cycle and given free access to food and water. *Borealin* ^+/−^ mice were obtained from wild-type (WT) and *Borealin* ^+/−^ mice. Embryos at each studied stage and male adults at 4 and 18 months of age were genotyped. Thyroids at embryonic stages from E13.5 to E17.5 and thyroids from adults aged 4 and 18 months were microdissected as described previously (17). At 18 months old, thyroid tissue of *Borealin* ^+/−^ mice had no Braf^V637E^ corresponding to BRAF^V600E^ in humans (three mice were investigated).

### Induction of hypothyroidism in adult mice

We used a well-validated model to induce hypothyroidism in 13 WT and 19 *Borealin* ^+/−^ male mice aged 4 months (18). The drinking water was supplemented with 0.02% methimazole (MMI; Sigma-Aldrich, St. Louis, MI, USA) and 0.5% sodium perchlorate (ClO_4_^−^) (Sigma-Aldrich) for 3 weeks.

### Assays on mouse serum samples

The serum was separated from blood samples collected from 4- and 18-month-old WT and *Borealin* ^+/−^ mice. Thyroid-stimulating hormone (TSH) was assayed using the Mouse Thyroid Stimulating Hormone (TSH) ELISA Kit (Abbexa, Cambridge, UK). Serum T4 was measured using an immunochemiluminescent assay using the Elecsys T4 assay on a cobas E801 (Roche Diagnostics, Basel, Switzerland). The assay was based on the recognition of the T4 by a sheep-origin antibody, in competition with a Ru2+-marked exogen T4. The assay measuring range was 5.4 to 320 nmol/L. Internal quality controls during the study indicated acceptable target values associated with a low coefficient of variation (CV < 5%).

### RNA extraction and quantitative real-time PCR

The thyroids were microdissected, snap-frozen immediately, and then stored at −80°C. Total RNA of sorted cells or thyroid tissue was isolated using the Qiagen RNeasy MicroKit or MiniKit (Qiagen, Valencia, CA, USA). The Maxima First Strand cDNA Synthesis Kit (Thermo Fisher Scientific, Waltham, MA, USA) was used for reverse transcription of 250 ng of each RNA sample. Each PCR was performed on 5 μL of synthesized cDNA diluted to 1/20 using the SybrGreen PCR Master Mix (Thermo Fisher Scientific) and primers. Peptidylprolyl isomerase A served as an endogenous control. Real-time PCR (RT-PCR) was performed using the QuantStudio 3 Real-Time PCR System (Thermo Fisher Scientific). The data were analyzed using the comparative cycle threshold method and reported as the fold change in gene expression, normalized for a calibrator of value 1. Primer sequences are listed in **Supplemental Figure G**.

### Immunohistochemistry and quantification

Mouse tissues were fixed by immersion in 3.7% buffered formalin and then embedded in paraffin. Subsequently, 4-μm-thick sections were mounted on StarFrost adhesive slides (Knittel Glaser, Braunschweig, Germany) and processed for immunohistochemistry, as previously described (17). The primary antibodies were used at the following dilutions: rabbit anti-BOREALIN (1:1,000, donated by William Taylor), rabbit anti-Nkx2-1 (1:2,500, #PA0100, BioPAT, Caserta, Italy), mouse anti-Ki67 (1:20, #550609, Becton Dickinson, Franklin Lakes, NJ, USA), mouse anti-BrdU (1/4, Amersham, Fairfield, CT, USA), mouse anti-TG (1:100, Dako-Cytomation, Glostrup, Denmark), mouse anti-I-Tg (1:500, donated by Carrie Ris-Stalpers), and rabbit anti-T4 (1:5,000, BioRad, Hercules, CA, USA). The fluorescent secondary antibodies were Alexa Fluor 594 goat anti-rabbit and Alexa Fluor 488 goat anti-mouse antibodies (1:400, Thermo Fisher Scientific). The nuclei were stained using the Hoechst 33,342 fluorescent stain (0.3 mg/ml; Thermo Fisher Scientific). Photographs were taken using a fluorescence microscope (Leitz DMRB; Leica, Wetzlar, Germany) and digitized using a chilled 3CCD camera (C5810; Hamamatsu Photonics, Hamamatsu City, Japan). The sections were then analyzed using ImageJ 1.32s (freeware, www.rsbweb.nih.gov/ij) as previously described (17, 19). The Nkx2-1-positive surface areas per section served to estimate the total thyroid surface area in μm^2^. For stained surface area quantification, we used alternate sections at E9.5 and E11.5, one of every three sections at E13.5 and E15.5, one of every four sections at E17.5, and one of every five sections in adults. The surface area values then served to estimate the total stained surface area for each thyroid and each marker: the average of all sections counted was compared to the total number of sections containing thyroid tissue. The proliferation of Nkx2-1-positive cells (at E9.5, E11.5, E13.5, and E17.5) was estimated by counting Ki67-positive nuclei among Nkx2-1-positive cells on alternate sections throughout the entire tissue sample. The surface areas positive for T4, a marker of advanced thyroid differentiation, were normalized for total thyroid surface area. At least five thyroids were analyzed per genotype. The results are reported as mean ± SEM.

For Nkx2-1 staining of adult mouse thyroid glands, the first immunohistochemistry steps were as described above. After application of the primary antibody, the sections were incubated with biotinylated secondary antibody for 1 h. Immunostaining was then performed using the Vectastain ABC Kit (Vector Laboratories, Burlingame, CA, USA) according to the manufacturer’s instructions. The sections were then incubated in 3,3′-diaminobenzidine tetrahydrochloride and counterstained with hematoxylin and eosin.

The surface area of follicles was estimated from serial transverse sections of thyroids from adult WT and *Borealin* ^+/−^ mice. The sections were deparaffinized and then stained with hematoxylin and eosin for quantification of follicles. Hematoxylin stains cell nuclei purple–blue, and eosin stains extracellular matrix and cytoplasm pink. Automated follicle size measurement was achieved using ImageJ 1.32s software. Follicles were classified by size as <1,000, 1,000–3,000, and >3,000 μm^2^ as previously described (20), and follicle size distribution was determined.

TUNEL experiments were performed using an *in situ* cell death detection kit (Roche, Neuilly-sur-Seine, France) according to the manufacturer’s instructions. Nkx2-1 immunostaining was then performed. To determine the percentage of apoptotic thyroid cells, the frequency of TUNEL-positive cells was counted among 300 Nkx2-1-positive cells.

### Microarray and analysis

After validation of the RNA quality with Bioanalyzer 2100 (using Agilent (Santa Clara, CA, USA) RNA 6000 nano chip kit),75 ng of total RNA was reverse transcribed following the GeneChip^®^ WT Plus Reagent Kit (Affymetrix, Santa Clara, CA, USA). Briefly, the resulting double-strand cDNA was used for *in vitro* transcription with T7 RNA polymerase. After purification according to Affymetrix protocol, 5.5 μg of Sens Target DNA was fragmented and biotin labeled. After control of fragmentation using Bioanalyzer 2100, cDNA was then hybridized to GeneChip^®^ Clariom S Mouse (Affymetrix) at 45°C for 17 h. After overnight hybridization, chips were washed on the fluidic station FS450 following specific protocols (Affymetrix) and scanned using the GCS3000 7G. The scanned images were then analyzed with Expression Console software (Affymetrix) to obtain raw data (cel files) and metrics for Quality Controls. Gene expression levels were calculated using the RMA (Robust Multichip Algorithm algorithm), and flags were computed using a custom algorithm within R (R Project for Statistical Computing http://www.r-project.org/). Assuming that a maximum of 80% of genes were expressed, the 20% lowest values were selected for each microarray as background. A threshold was fixed at two standard deviations over the mean of the background. All probes whose normalized intensity measures were lower than the computed threshold were flagged 0 instead of 1. Three probe lists were used for each comparison according to flagged measurements in the relevant chips. The “P50” list had been created filtering probes flagged as “Present” for at least half of the chips. The group comparisons were performed using Student’s t-test. To estimate the false discovery rate, the resulting *p*-values at 5% were filtered, and the Benjamini and Hochberg, Bonferroni, or without correction was used. Cluster analysis was performed by hierarchical clustering using Spearman’s correlation similarity measure and average linkage algorithm.

Data were submitted to Ingenuity Pathway Analysis (IPA http://www.ingenuity.com) and Gene Set Enrichment Analysis (GSEA https://www.gsea-msigdb.org/gsea/index.jsp) to model networks and unveil relevant pathways and biological processes.

### Western blotting studies of mouse thyroid tissue

Proteins from mouse thyroid tissue collected in radioimmunoprecipitation assay (RIPA) buffer and sonicated were quantified using the bicinchoninic acid (BCA) protein assay (Thermo Fisher Scientific). Then, 20 μg of total protein was separated on Bis–Tris polyacrylamide gel with a 4%–12% gradient (Thermo Fisher Scientific) and transferred onto polyvinylidene difluoride (PVDF) membranes (Thermo Fisher Scientific). The membranes were incubated with the primary antibodies rabbit anti-P-ERK, ERK, (1:1,000, #4370S, 9102S, Cell Signaling Technology, Danvers, MA, USA) or rabbit anti-Vinculin (1:1,000, #4650, Cell Signaling) and then with horseradish peroxidase-conjugated goat anti-rabbit antibody. The binding of secondary antibodies was revealed using the Amersham ECL Prime Detection Reagent Kit (GE Healthcare, Chicago, IL, USA). The protein bands on the membranes were scanned with the ImageQuant LAS 4000 Station (GE Healthcare) and then analyzed using ImageJ 1.32s to determine the protein levels.

### Statistics

The sample size was estimated based on previous experience with similar studies (17, 21). Results are reported as mean ± SEM for the number of experiments indicated in the figure legends. Statistical analyses were performed using GraphPad Prism4 (GraphPad, La Jolla, CA, USA). Comparisons were with the unpaired Mann–Whitney test or Fisher’s test, as indicated in the figure legends. Differences were considered significant when *p* < 0.05.

## Results

### Borealin expression in mouse thyroid

**Figure 1:**
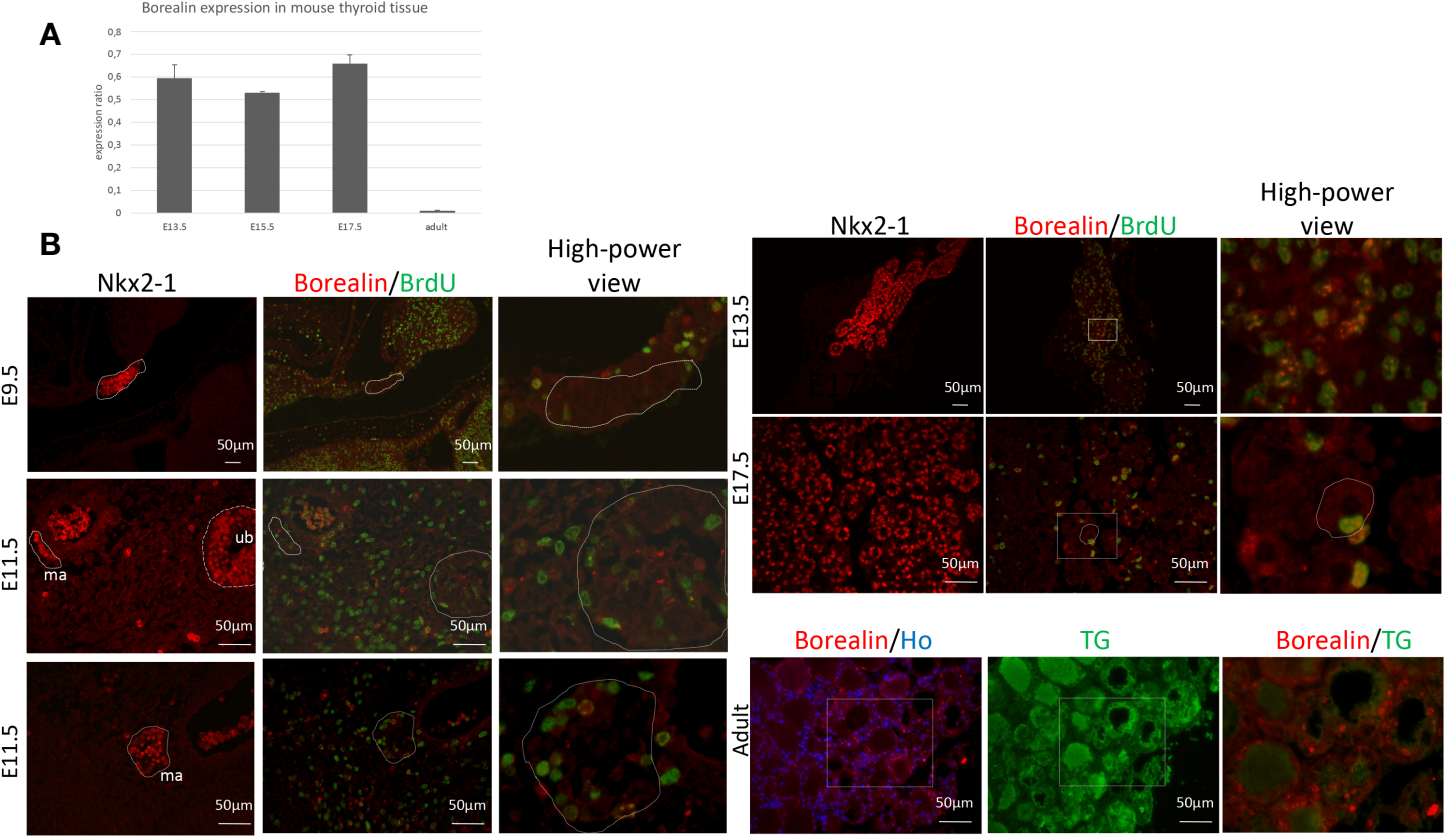
Borealin expression in mouse thyroid tissue: high in embryos and low in adults. **(A)** Quantitative PCR assessment of *Borealin* expression by thyroid tissue on embryonic days E13.5, E15.5, and E17.5, and, in adulthood, normalized for thyroid tissue on E13.5 and peptidylprolyl isomerase **(A)** Two tissue samples (pool of three to five thyroids) were studied at each developmental stage. **(B)** Immunohistofluorescence staining for Nkx2-1 (in red) and co-staining for Borealin (in red) and BrdU (in green) in whole embryos on E9.5 and E11.5 and in thyroid tissue on E13.5 and E17.5; on E11.5, the median anlage (ma) and ultimobranchial bodies (ub) can be identified. In adults, thyroid samples were co-stained for Borealin (in red) and thyroglobulin (TG, in green). Hoechst stained nuclei in blue. High-power views of the surrounding regions are provided in the right-hand columns. E, embryonic day; Nkx2-1, Nkx2 Homeobox 1; BrdU, bromodeoxyuridine; TG, thyroglobulin; ma, midline anlage; ub, ultimobranchial body; Ho, Hoechst.

We found intense Borealin expression during development and lower expression levels in adulthood using quantitative PCR (qPCR) on WT thyroids (**Figure 1A**). In mouse embryos, Borealin protein was detectable by immunohistofluorescence in the nuclei of a few thyroid anlage cells at E9.5 and the midline anlage and ultimobranchial bodies on E11.5 (**Figure 1B**). Interestingly, in ultimobranchial bodies at E11.5, Borealin was observed in progenitor cells during mitosis. Borealin was found at E13.5 in the nuclei of Nkx2-1-expressing cells, i.e., chiefly thyrocyte progenitors and, on E17.5, in the thyrocytes (**Figure 1B** and **Supplemental Figure A**). During development, Borealin was co-expressed in some bromodeoxyuridine (BrdU)-positive (proliferating) cells. In adults, Borealin was very scantly expressed in the thyrocyte nuclei of thyroid tissue except in some regions (**Figure 1B**).

### Thyroid phenotype of *Borealin* ^+/−^ mice

#### Thyroid development was abnormal from E9.5 onward

**Figure 2:**
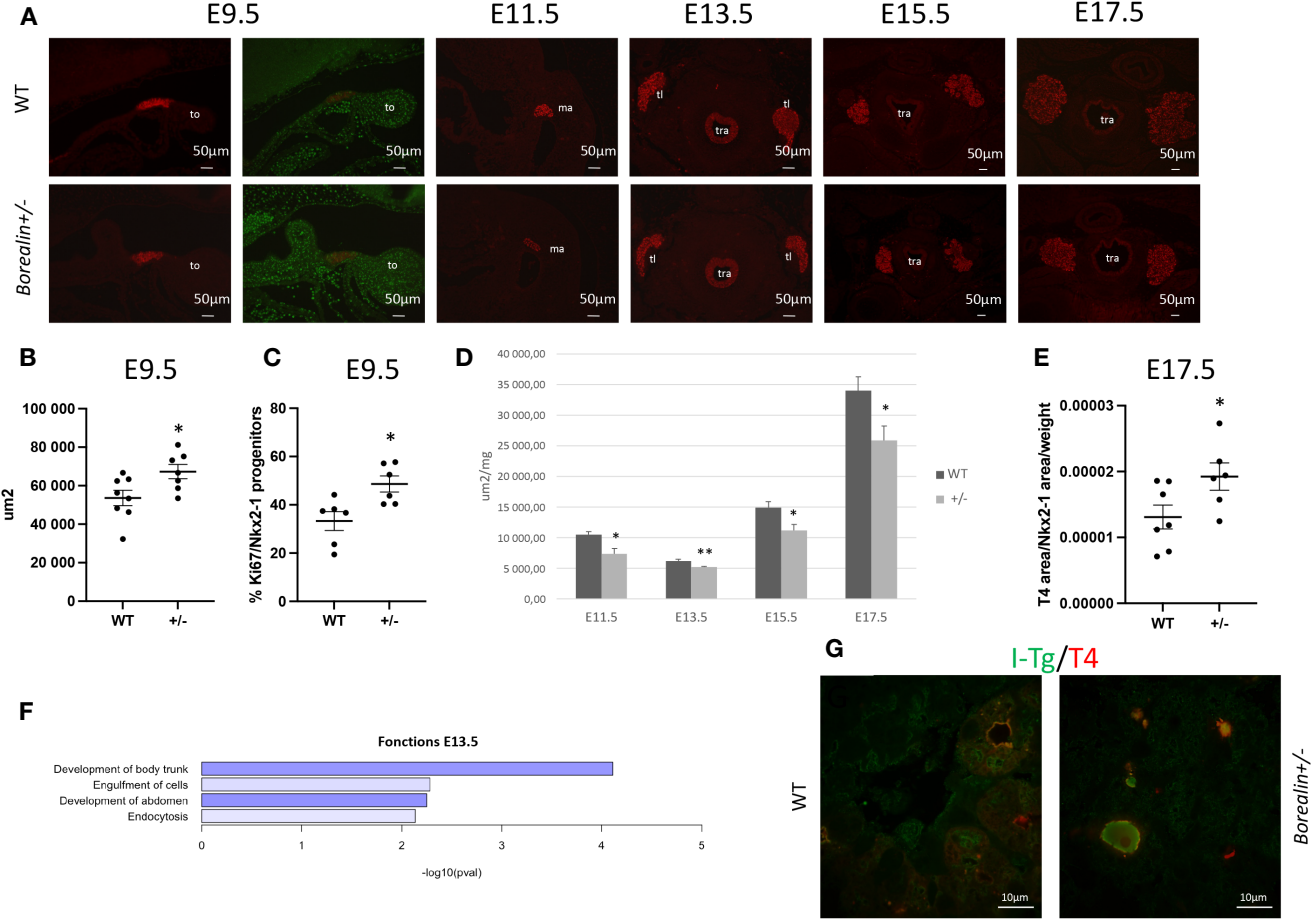
Abnormal thyroid development in *Borealin* ^+/−^ *mice*. **(A)** Thyroid morphology was investigated using Nkx2-1 staining on E9.5 and E11.5 (sagittal sections) and E13.5, E15.5, and E17.5 (transverse sections) in wild-type (WT) and *Borealin* ^+/−^ littermates. Nkx2-1 is a marker of early thyrocyte progenitors and differentiated thyrocytes. Nkx2-1 is stained in red, and the proliferation marker Ki67 is in green. Note an increase of thyroid anlage at E9.5 in the *Borealin* ^+/−^ mice than in the WT mice. WT, wild type; to, tongue; ma, median anlage; tl, thyroid lobe; tr, trachea. **(B)** Total thyroid surface area (μm^2^) quantified by Nkx2-1 staining on E9.5 was larger in the *Borealin* ^+/−^ mice than in the WT mice. Seven to eight mice with each genotype were investigated. **(C)** Proliferation ratio calculated as the proportion of Nkx2-1-positive cells labeled with Ki67 on E9.5 (as described in Material and Methods): thyroid anlage hyperplasia in Borealin^+/−^ *vs.* WT mice. Six mice with each genotype were investigated. **(D)** Total thyroid surface area (μm^2^) was quantified using Nkx2-1 staining on E11.5, E13.5, E15.5, and E17.5, with normalization for the weight of each embryo. At each of the four stages and for each genotype, five to seven mice were studied. Note that from E11.5 onward, the *Borealin* ^+/−^ thyroids were smaller (black bars) compared to the WT thyroids (gray bars). **(E)** Ratio of T4-positive surface area over Nkx2-1-positive surface area normalized for embryo weight at E17.5. Six to seven mice with each genotype were investigated. Note the larger amount of T4 in the *Borealin* ^+/−^ *vs.* the WT thyroids. **(F)** Transcriptomic analysis of thyroid tissue on E13.5. The genes with lower expression levels in the *Borealin* ^+/−^ mice *vs.* the WT mice were involved in body trunk development, cell engulfment, abdomen development, and endocytosis. Genes with lower expression were in blue. **(G)** Co-staining for I-Tg and T4 in the *Borealin* ^+/−^ *vs.* the WT thyroids at E17.5. Note the increased staining of Tg-I surface area in the *Borealin* ^+/−^ *vs.* the WT thyroids. The data are mean ± SEM. *p < 0.05 and **p < 0.01, Mann–Whitney test. E, embryonic day; WT, wild type; Tg, thyroglobulin; I-Tg, iodinated thyroglobulin.

We used immunohistochemistry to detect Nkx2-1, a marker for progenitor cells and thyrocytes. At E9.5, the midline anlage appeared thicker, and the thyroid-anlage surface area was 25% larger (*p* < 0.05) in the *Borealin* ^+/−^ *vs.* WT mice (**Figures 2A, B**). Indeed, the number of cells in the median anlage at E9.5 was increased by 32% in *Borealin* ^+/−^ *vs.* WT mice (**Supplemental Figure B**). This size increase was ascribable to increased proliferation of Nkx2-1-expressing progenitors: the proliferation ratio evaluated using a nuclear proliferation marker Ki67 was 46% higher in *Borealin* ^+/−^ mice than in WT mice (*p* < 0.05) (**Figure 2C**). Nkx2-1 staining showed thyroid anlage fragmentation in two of nine *Borealin* ^+/−^ embryos *vs.* none of eight WT embryos (**Supplemental Figure C**).

Between E11.5 and E17.5, thyroid surface area was significantly smaller in *Borealin* ^+/−^ *vs.* WT mice, with decreases of 30% on E11.5, 15% on E13.5, 25% on E15.5, and 25% on E17.5 (*p* < 0.05 for all four comparisons) (**Figures 2A, D**). On E11.5, the midline anlage and ultimobranchial bodies exhibited similar decreases in surface area. Cell proliferation as assessed using Ki67 did not differ significantly between groups on E11.5, E13.5, or E17.5 (**Supplemental Figure D**), whereas on E13.5 and E17.5, the proliferation ratio tended to be higher in the *Borealin* ^+/−^ group than in the WT group. Apoptosis on E11.5 and E13.5 was not significantly different between the two groups. The transcriptomic analysis of E13.5 thyroids from *Borealin* ^+/−^ and WT thyroids evidenced negative enrichment of genes involved in engulfment, endocytosis, and development of the abdomen and body trunk (**Figure 2F**). The qPCR assays performed on E15.5 and E17.5 samples to assess the thyroid markers Nkx2-1, Pax8, and Foxe1 (transcription factors) and differentiation markers such as Tg, Tpo, and Nis showed no significant differences between *Borealin* ^+/−^ and WT thyroids (**Supplemental Figure E**).

T4 staining showed a significant increase in the ratio of T4-positive over total thyroid surface area and embryo weight in *Borealin* ^+/−^ compared to WT mice at E17.5 (**Figure 2E**). The amount of I-Tg stored in the follicles was greater in the *Borealin* ^+/−^ mice than in the WT mice at E17.5 (**Figure 2G**).

Thus, *Borealin* invalidation led to abnormal thyroid size during development from thyroid budding to maturation.

#### At 4 months old, the Borealin^+/−^ mice developed a goiter with large follicles

**Figure 3:**
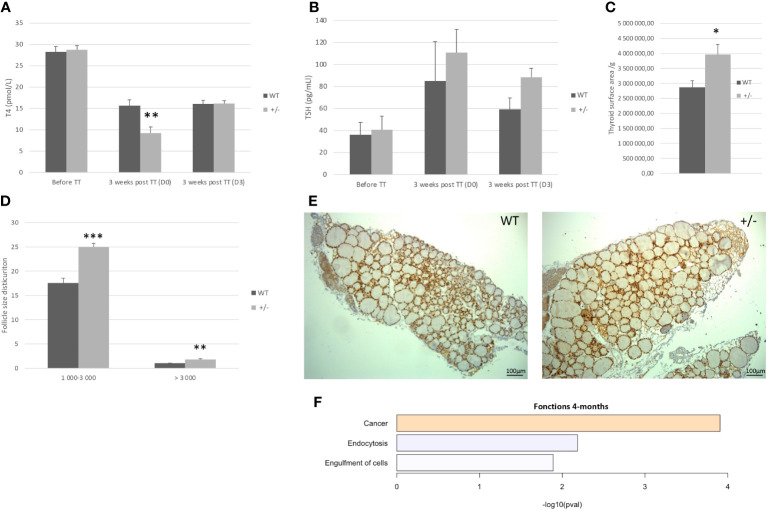
Four-month-old mice: *Borealin* ^+/−^ thyroids are hypertrophic, with large follicles. **(A, B)** Serum T4 **(A)** and thyroid-stimulating hormone (TSH) **(B)** levels in male mice given antithyroid drugs for 3 weeks; 19 *Borealin* ^+/−^ and 13 wild-type (WT) mice were investigated for T4 assay and three to 13 mice for TSH assay. Assays were performed before treatment and then at treatment discontinuation (D0) and 3 days later (D3). Note the increased sensitivity of the *Borealin* ^+/−^ mice to the treatment, with 45% and 68% less T4 on D0 (*p* < 0.01) and 134% and 172% more TSH on D0 compared to the WT (black bars) and *Borealin* ^+/−^ (gray bars) mice. **(C)** Thyroid surface area estimated by Nkx2-1 staining normalized for body weight. Note the significantly larger thyroids in *Borealin* ^+/−^ mice *vs.* WT mice (*p* < 0.05). Five mice with each genotype were investigated. **(D)** Follicles were divided into two size categories based on whether the lumen surface area was 1,000–3,000 μm^2^ or >3,000 μm^2^. Four to six animals per genotype were investigated. **(E)** Nkx2-1 staining (in brown) of thyroid tissue from 4-month-old mice. Note the larger thyroids and larger follicles in *Borealin* ^+/−^ mice *vs.* WT mice. **(F)** Transcriptomic analysis of thyroid tissue from 4-month-old mice (three per genotype). The genes with lower expression levels in the *Borealin* ^+/−^ mice *vs.* the WT mice were involved in endocytosis and cell engulfment (in blue). The genes with higher expression levels in the *Borealin* ^+/−^ mice *vs.* the WT mice were involved in tumorigenesis (in orange). The data are mean ± SEM. *p < 0.05, **p < 0.01, and ***p < 0.001, Mann–Whitney test. E, embryonic day; WT, wild type; TT, treatment.

At 4 months of age, *Borealin* ^+/−^ mice had normal thyroid function as assessed by plasma TSH and T4 levels (**Figures 3A, B**). The thyroids were 38% larger in the *Borealin* ^+/−^ group *vs.* the WT group (*p* < 0.01) (**Figure 3C**). The follicles were also significantly larger in the *Borealin* ^+/−^ group (*p* < 0.01) (**Figures 3D, E**).

For further investigation of thyroid function, we induced hypothyroidism using a validated methodology by administering an antithyroid drug (methimazole) and sodium perchlorate for 3 weeks to male *Borealin* ^+/−^ and WT mice. We assayed serum T4 and TSH at treatment initiation and then at treatment discontinuation (D0) and 3 days later (D3). Features of hypothyroidism including patchy alopecia and dry skin developed in both groups but were more marked in the *Borealin* ^+/−^ mice. T4 on D0 *vs.* before treatment was 68% and 45% lower in the *Borealin* ^+/−^ and WT groups (*p* < 0.01), while TSH was 172% and 134% higher, respectively. On D3, T4 was higher and TSH was lower than those on D0.

Transcriptomic analysis of thyroids from both groups showed decreases in genes involved in endocytosis and engulfment in the *Borealin* ^+/−^ group (**Figure 3F**), as well as an increase in genes involved in cancer function.

Thus, 4-month-old *Borealin* ^+/−^ mice had normal thyroid function, enlarged thyroids and follicles, increased sensitivity to antithyroid drugs, and transcriptome abnormalities.

#### At 18 months old, the Borealin^+/−^ mice developed a goiter with abnormal structures

Based on the role of Borealin during mitosis and for thyroid physiology (12, 14), we followed the evolution of the thyroid during old age.

**Figure 4:**
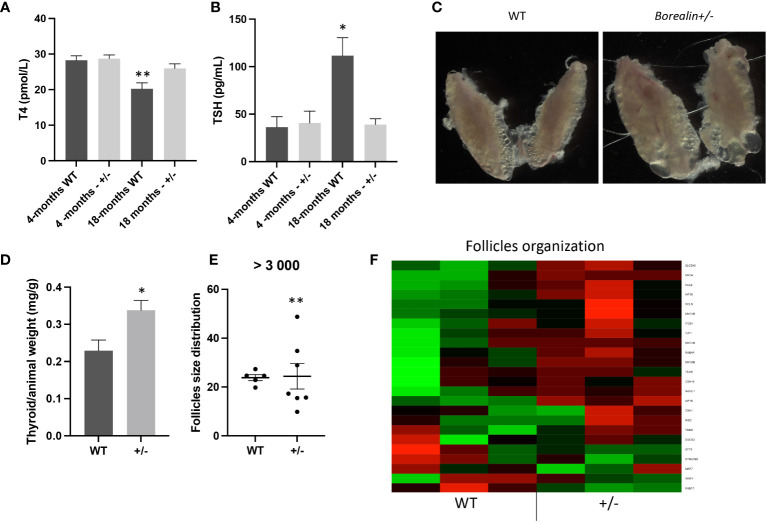
Eighteen-month-old mice: *Borealin* ^+/−^ thyroids are hypertrophic, with abnormal follicles. **(A, B)** Serum T4 levels were higher **(A)** and serum thyroid-stimulating hormone (TSH) levels lower **(B)** in *Borealin* ^+/−^ *vs.* wild-type (WT) mice (six to 10 with each genotype); the Borealin-deficient mice had normal thyroid function, whereas the WT mice developed hypothyroidism. Assays at 4 months and 18 months were added for better understanding. **(C)** Abnormal thyroid morphology with variable follicle size in *Borealin* ^+/−^ mice. **(D)** Thyroid weight normalized for body weight (10 WT and eight *Borealin* ^+/−^ mice). Note the significant hyperplasia of the *Borealin* ^+/−^ thyroids. **(E)** Follicle size distribution among follicles >3,000 μm² (five to seven mice with each genotype). Note the significantly greater size heterogeneity in the *Borealin* ^+/−^ group. **(F)** Heatmap of genes involved in thyroid follicle organization (three mice with each genotype). Note the marked overexpression of these genes in the *Borealin* ^+/−^ group compared to the WT group. The genes with low expression are in green, and the genes with high expression are in red. The data are mean ± SEM. *p < 0.05, **p < 0.01, and ***p < 0.001, Mann–Whitney test, Fisher’s test. WT, wild type.

**Figure 5:**
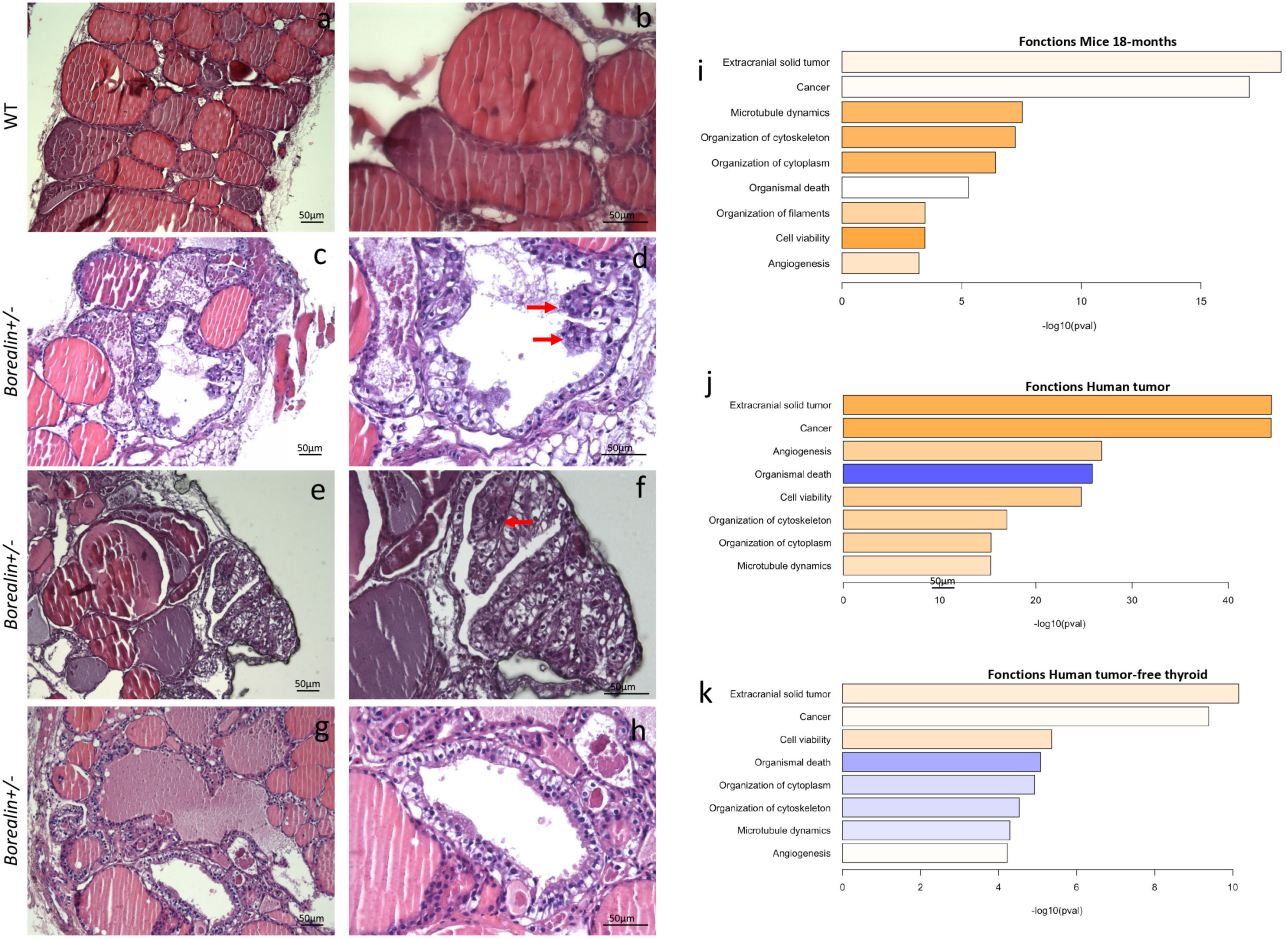
Abnormal papillary-like structures and cytoskeleton defects in the *Borealin* ^+/−^ thyroids at 18 months; comparison with thyroid tissue from a human with a heterozygous *BOREALIN* mutation. **(A–H)** Hemalum-eosin-stained thyroid tissue from *Borealin* ^+/−^ **(C–H)** and wild-type (WT) **(A, B)** mice. **(A, B)** Normal thyroid architecture with colloid in follicles. **(C, D)** Large increase in follicle size and a loss of colloid with some papillary structure (see arrow). **(E, F)** Enlarged follicles containing some papillary structures (see arrow). **(G, H)** Enlarged follicles without colloid with higher cells without nuclear atypia. Note the thyroid tissue changes in the *Borealin* ^+/−^ sample, which contains papillary-like structures and cells with abundant cytoplasm surrounding empty follicles. On the right panel, high-power view of specific regions. **(I)** Transcriptomic analysis of mouse thyroid tissue at 18 months. The genes with higher expression levels in the *Borealin* ^+/−^ mice *vs.* the WT mice are involved in extracranial tumor, cancer, microtubule dynamics, cytoskeleton organization, and filament organization. **(J)** Transcriptomic analysis of the papillary thyroid carcinoma from the patient with a heterozygous *BOREALIN* ^+/−^ mutation and *BRAF*-mutated thyroid tumor. The genes with high expression levels include those highly expressed in the *Borealin* ^+/−^ mice. **(K)** Transcriptomic analysis of tumor-free thyroid tissue from the patient with a heterozygous *BOREALIN* ^+/−^ mutation and papillary thyroid carcinoma. The genes involved in cytoskeleton are decreased in the tumor-free thyroid tissue of the patient.

Plasma T4 and TSH levels were 27% higher and 65% lower, respectively, in *Borealin* ^+/−^ mice *vs.* WT mice (*p* < 0.01 and *p* < 0.05, respectively) (**Figures 4A, B**). Thyroid weight normalized for body weight was 47% higher in the *Borealin* ^+/−^ mice *vs.* the WT mice (*p* < 0.01) (**Figures 4C, D**). The T4 and TSH values in the *Borealin* ^+/−^ group were within the normal range (euthyroid goiter), whereas the WT mice had hypothyroidism. In the *Borealin* ^+/−^ group, follicle size distribution was significantly more heterogeneous (Fisher’s test, *p* < 0.01) (**Figure 4E**), and the thyroids contained very large follicles and nodular structures (**Figure 4C**). Hematoxylin and eosin staining, a common method for viewing cellular and tissue structure, revealed modified follicles and microfoci with papillary structures in the *Borealin* ^+/−^ mice but not in the WT mice (**Figures 5A–H**). Thyrocytes had a normal aspect, but there was a large increase in follicle size and a loss of colloid in *Borealin* ^+/−^ thyroids (**Figures 5C, D**). We also observed foci of hyperplastic cells with abundant cytoplasm (**Figures 5C, D, G, H**) and microfoci of cells displaying a characteristic papillary structure (**Figures 5C–F**). However, we did not find nuclear inclusions and cleared nuclei, which are characteristic features of human PTC. The transcriptomic analysis showed marked differences in genes involved in follicle organization between thyroids of *Borealin* ^+/−^ and WT mice (**Figure 4F**). This gene set was inspired by Koumarianou et al. as genes involved in apical–basal thyroid cell polarization, lumen formation, and follicle maturation (22).

#### Borealin, thyroid cancer in mice and in a subject with a BOREALIN mutation

**Figure 6:**
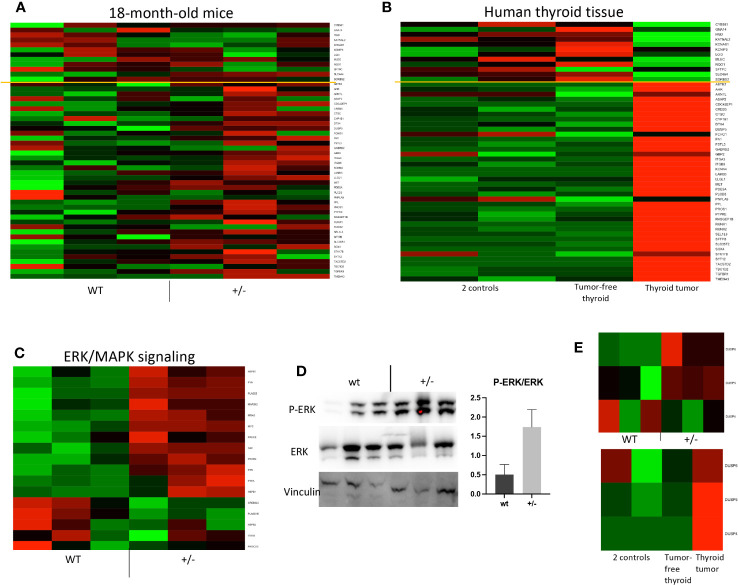
*Braf*-associated gene expression and ERK pathway activation in *Borealin* ^+/−^ thyroids. **(A)** Transcriptome of 71 genes in *Borealin* ^+/−^ and wild-type (WT) thyroid tissue. The Cancer Genome Atlas Research network used 391 human papillary thyroid cancer samples to identify 71 genes, which then served to derive a score differentiating *BRAF* ^V600E^ *-*driven from *RAS-*driven tumors limited by the yellow line (9). The transcriptome results support Braf^V600E^-associated gene expression in the *Borealin* ^+/−^ thyroids. The genes with low expression at the top are in green, and all the genes with high expression at the bottom are in red. **(B)** Same analysis on samples from the patient with a heterozygous *BOREALIN* ^+/−^ mutation and papillary thyroid carcinoma. The tumor is clearly *BRAF*-driven. Note the marked differences between the tumor-free thyroid tissue from this patient and thyroid tissue from two controls. **(C)** Heatmap of ERK/MAPK signaling in thyroid tissue from *Borealin* ^+/−^ and WT mice. Note the increased expression of genes involved in ERK/MAPK signaling in the *Borealin* ^+/−^ thyroids. **(D)** Western blotting of P-ERK, ERK, and vinculin, with quantification of the P-ERK/ERK ratio for *Borealin* ^+/−^ and WT thyroids. Note the P-ERK increase in the *Borealin* ^+/−^ thyroids. **(E)** DUSP6, DUSP5, and DUSP4 mRNAs in thyroid tissue. Top: *Borealin* ^+/−^ and WT mice; note the increased expression of DUPS6 and DUPS5 in the *Borealin* ^+/−^ thyroids. Bottom: DUSP6, DUSP5, and DUSP4 mRNAs in thyroid tissue from two controls and in tumor-free thyroid tissue and thyroid tumor from the patient with a heterozygous *BOREALIN* ^+/−^ mutation; note the high DUSP expression in the tumor.

We performed transcriptomic analyses of thyroid tissue samples from 18-month-old *Borealin* ^+/−^ and WT mice. Compared to the WT mice, the *Borealin* ^+/−^ mice exhibited positive enrichment in expressed genes involved in malignancy function (extracranial solid tumor and cancer), cytoskeletal and/or cytoplasmic functions (microtubule dynamics and organization of the cytoskeleton, filaments, and cytoplasm), viability (increased cell viability and decreased organismal death), and angiogenesis (**Figure 5I**). Then, we deciphered the thyroid abnormalities observed in *Borealin* ^+/−^ mice. Strong activation of *Braf*-like genes described by The Cancer Genome Atlas (TCGA) network of PTC (9) was found in the *Borealin* ^+/−^ thyroids (**Figure 6A**). ERK/MAPK signaling was also activated (**Figure 6C**), and P-ERK protein expression increased (**Figure 6D**) in the *Borealin* ^+/−^ *vs.* WT thyroids. DUSP5 and DUSP6 expression levels were higher in the *Borealin* ^+/−^ thyroids than in the WT thyroids (**Figure 6E**). These data showed overexpression of tumorigenesis genes and MAPK pathway activation in *Borealin* ^+/−^ thyroids.

We previously reported that the mother of a girl with TD and CH had normal thyroid function, asymmetrical thyroid lobes, and PTC (12). She and her daughter were heterozygous for the c.341G>A, p.R114Q *BOREALIN* mutation. The tumor cells carried the *BRAF* ^V600E^ mutation. By whole exome sequencing, no additional *BOREALIN* variant was observed in the thyroid tumor part, and *BOREALIN* expression was equivalent between thyroid tumor and tumor-free thyroid by transcriptomic analysis. In the patient, gene sets of the cytoskeleton and cytoplasmic functions were increased in the thyroid tumor (**Figure 5J**) as in *Borealin* ^+/−^ thyroids and decreased in the tumor-free thyroid (**Figure 5K**). Angiogenesis genes and cell viability were increased and organismal death decreased in two thyroid regions with and without tumor (**Figures 5J, K**) as in *Borealin* ^+/−^ thyroids. The tumor signaling pathways (extracranial solid tumor and cancer pathway) were activated in the *Borealin* ^+/−^ mouse thyroids (**Figure 5I**) and the tumoral and tumor-free thyroid samples from the patient, with a very high ratio in the tumoral human tissue. Strong activation of *Braf*-like genes was found in the tumor as in *Borealin* ^+/−^ thyroids, but not the tumor-free tissue, from the human patient (**Figure 6B**). DUSP5 and DUSP6 expression levels were higher in human thyroid tumors than in the tumor-free tissue and controls (**Figure 6E**).

The Braf-like signature observed in *Borealin* ^+/−^ mice’s thyroid tissue, structures, and transcriptomic analysis was in line with the patient’s tumor.

## Discussion

Borealin-deficient mice had abnormal thyroid development, thyroid goiter with follicle disorganization at 4 months of age, and further structural thyroid abnormalities as papillary tumor-like structures at 18 months. Transcriptome abnormalities were evidenced both in the *Borealin* ^+/−^ mice and in PTC tissue from a human patient carrying a *BOREALIN* mutation. These findings establish a role for Borealin in thyroid development, function, and tumorigenesis.

The thyroid anlage was hyperplastic in *Borealin* ^+/−^ mice on E9.5. Normally, progenitor cells show very little proliferation on E9.5, as shown in our WT mice. The proliferation ratio was accurately regulated in thyroid anlage, and any abnormality disturbs thyroid development afterward. Disrupted thyroid development after the demonstration of an increased proliferation ratio on E9.5 has been reported (21). Defects of endocytosis observed at E13.5 by transcriptomic analysis could lead to defects of TG-colloid engulfment at E17.5 with high thyroid T4 content seen in the *Borealin* ^+/−^ mice. Consequently, *Borealin* ^+/−^ mice displayed hypoplastic thyroid from E11.5 and more intra-thyroid T4 later in development.

At 4 months old, *Borealin* ^+/−^ thyroids were hyperplastic with large disorganized follicles. One hypothesis is gland enlargement to compensate for insufficient T4 release from the gland, possibly related to impaired TG endocytosis. The transcriptomic analysis showing decreases in genes involved in endocytosis and engulfment supports this hypothesis at 4 months old. Endocytosis and engulfment are processes involved in TG internalization via vesicle-mediated endocytosis at the apical membrane of thyrocytes for TH secretion at the basal membrane (23, 24). Decreased TG endocytosis would also explain the follicle enlargement seen in the *Borealin* ^+/−^ mice (25). The consequence of follicle defects would be an unadapted response to antithyroid drugs leading to more profound hypothyroidism at 4 months old. Due to their deficiency in releasing T4, *Borealin* ^+/−^ thyroids may not adapt to stress and respond to a specific increased need for TH. Thus, Borealin plays a role in proper thyroid hormone synthesis and release and also in homeostasis. The thyroid hyperplasia with large disorganized follicles persisted from adulthood to older age. At 18 months old, *Borealin* ^+/−^ mice had structural follicular defects of thyroid tissue with an increase of follicular polarization gene sets (22). In addition to that, most of these genes are linked to cytoskeleton organization. *Borealin* ^+/−^ thyroids were enriched in cytoskeleton gene sets compared to WT thyroids. Borealin binds to the microtubules, and Borealin deficiency may therefore alter the thyrocyte cytoskeleton, explaining the abnormal follicle shapes in the *Borealin* ^+/−^ mice (26). Thus, Borealin is involved not only in the CPC, which controls mitosis, but also in cell and tissue structure.

In addition, despite heterogeneous and disorganized follicles, *Borealin* ^+/−^ mice still have normal thyroid function at this advanced age. In contrast, thyroid function is decreased in the 18-month-old WT mice with larger surface area and larger follicles in comparison with 4-month-old mice (**Supplemental Figure F**). Similarly, in a study of healthy mice, the thyroid follicles at 23 months old were larger, plasma TSH levels were higher, and plasma T4 levels were lower than at 3 months old, consistent with the development of aging-related hypothyroidism (27). At 18 months old, due to their old age, WT and *Borealin* ^+/−^ mice had larger follicles and larger thyroid than at 4 months old, but only WT mice had hypothyroidism. The WT mice had a decrease in thyroid activity due to their age, whereas *Borealin* ^+/−^ mice had persistent thyroid activity leading to an undeclined normal thyroid function. This is probably why we observed by thyroid transcriptomic analysis an enrichment in angiogenesis, cellular viability gene sets, and a decrease in organismal death when compared to WT thyroids. We hypothesize that to maintain thyroid homeostasis, the activity of the gland should be stimulated. This regulatory mechanism encouraged in a sustained manner can lead to tissue changes favoring tumorigenesis. Thus, Borealin plays a role in thyroid homeostasis.

At an older age, *Borealin* ^+/−^ thyroids were enriched in cancer gene sets compared to WT thyroids, which may be due to defects of homeostasis with hyperactivity of the gland. Older *Borealin* ^+/−^ mice exhibited activation of *Braf*-associated genes when compared to WT mice, further supporting a link between genes for Borealin and BRAF pathway. Moreover, the microfoci seen in aged *Borealin* ^+/−^ mice had a papillary and vesicular structure consistent with *Braf*-driven tumorigenesis (28). Also, ERK/MAPK signaling activation, a known effect of the oncogenic *BRAF* mutation, was found in *Borealin* ^+/−^ thyroids (10). Thus, the *Borealin* deficiency in thyroids activated the ERK/MAPK signaling leading to disorganization of tissue with papillary-like structures. Indeed, we previously described data from an adult with thyroid dysgenesis and a heterozygous *BOREALIN* mutation (c.341G>A, p.R114Q) who developed PTC (12). The tumor cells harbored the *BRAF* ^V600E^ mutation known to be associated with PTC (10) and presented enrichment of genes involved in cancer and BRAF signature like the thyroid of *Borealin* ^+/−^ mice. Borealin has been reported to be involved in the development of several types of cancer in humans, including breast cancer, cutaneous melanoma, and lung cancer (29). A study of human lung cancer samples demonstrated that overexpression of the Borealin was associated with a poor prognosis (29). In TCGA database of human PTC, *BOREALIN* expression correlated with tumor dedifferentiation (**Supplemental Figure H**). As we observed papillary tumor-like structure in thyroid mice with Borealin deficiency, we supposed that Borealin is tightly regulated with a narrow frame of gene expression level. At 18 months old, *Borealin* ^+/−^ mice have normal TSH but developed thyroid tumor-like, suggesting that the lack of Borealin under normal TSH stimulation at the late stage induces larger and later tumor-like development such as in other thyroid mouse models (22, 30). In addition, this may be in relation to what has been reported in humans harboring variants in genes implicated in TH synthesis (*NIS*, *TG*, *TPO*, and *SLC26A4*); thyroid goiter with dyshormonogenesis may develop thyroid cancer later in life (31). Thus, impairment in the expression or function of any protein involved in thyroid development or hormonogenesis can lead to thyroid tumor predisposition.

The thyroid phenotype differed between the *Borealin* ^+/−^ mice and the patients carrying *BOREALIN* mutations in our previous study (12). These last exhibited ectopic thyroid, athyreosis, hemi-agenesis, or thyroid asymmetry, all of which were absent in the Borealin-deficient mice but also difficult to observe in mice. However, identical phenotypes between humans and mice were very rare (32, 33). Thyroid function in the patients was normal or deficient (12). Mutations carried by patients are localized in a domain with no known function between two crucial domains for mitosis. In transgenic mice, *Borealin* ^+/−^, all the domains are deleted in a heterozygous manner. Our Borealin-deficient mice had large thyroids with large, disorganized follicles, a high T4 content, and normal thyroid function but increased susceptibility to antithyroid drugs. The patient whose thyroid tumor we studied here had normal thyroid function, whereas her daughter, who carried the same Borealin mutation, had CH, suggesting incomplete penetrance in humans. A major similarity between the Borealin-deficient mice and the human patient is the presence in thyroid cancer tissue of a *BRAF*-associated signature. The respective contributions of the *BOREALIN* and *BRAF* mutations to tumor development in the patient cannot be determined from our data. Nonetheless, close monitoring for thyroid cancer of patients carrying *BOREALIN* mutations seems warranted.

In conclusion, our results demonstrate a key role for Borealin in thyroid development and function. Despite being expressed at high levels only during early development, Borealin may have a role throughout life for thyroid tissue architecture and homeostasis. Borealin deficiency may increase the risk of thyroid tumorigenesis.

## Data availability statement

The raw data supporting the conclusions of this article will be made available by the authors, upon reasonable request. Requests to access the datasets should be directed to AC (aurore.carre@inserm.fr) or MP (michel.polak@aphp.fr).

## Ethics statement

The studies involving humans were approved by the French institutional review board. The studies were conducted in accordance with the local legislation and institutional requirements. The participants provided their written informed consent to participate in this study. The animal study was approved by Direction Departementale de la Protection des Populations for the French Ministry of Research, Health and Agriculture (Paris) under agreement number A75-13–19 in accordance with approved guidelines of French and European legislation. The study was conducted in accordance with the local legislation and institutional requirements. Written informed consent was obtained from the individual(s) for the publication of any potentially identifiable images or data included in this article.

## Author contributions

HD-M: Formal Analysis, Investigation, Writing – original draft. AS: Investigation, Writing – review & editing. DK: Writing – review & editing. SY: Investigation, Writing – original draft. BC-P: Formal Analysis, Writing – review & editing. LG: Writing – review & editing. FS: Writing – review & editing, Resources. NC: Formal Analysis, Software, Writing – review & editing. PN: Formal Analysis, Software, Writing – review & editing. DL: Writing – review & editing. MP: Writing – review & editing, Conceptualization, Funding acquisition, Supervision. AC: Conceptualization, Funding acquisition, Supervision, Writing – review & editing, Data curation, Formal Analysis, Investigation, Methodology, Writing – original draft.

## Supplementary material

Supplementary Figure
**(A)** Immunohistofluorescence staining for Borealin (in red) and BrdU (in green) in thyroid tissue on E13.5 and E17.5. Hoechst stained nuclei in blue. Note the Borealin expression in nuclei of thyrocytes progenitors and thyrocytes (co-staining Borealin/Hoescht/BrdU). **(B)** Number of cells Nkx2-1-positive in median anlage at E9.5. Note the increase of number of progenitors in *Borealin* ^+/^ *^-^* compared to WT. *P<0.05*. **(C)** Thyroid morphology using Nkx2-1 staining on E9.5 (sagittal sections) of *Borealin* ^+/-^ littermate. Note thyroid anlage fragmentation at E9.5 in this *Borealin* ^+/-^ embryo. to: tongue. **(D)**. Proliferation ratio calculated as the proportion of Nkx2-1-positive cells labelled with Ki67 on E11.5, E13.5 and E17.5: no significant difference. Three to four tissue samples were studied at each developmental stage. **(E)** Quantitative PCR assessment of thyroid markers expression by thyroid tissue on embryonic days E15.5 and E17.5 normalized for peptidylpropyl isomerase A and WT. Thyroid markers: Foxe1, Nkx2-1, Pax8, Tg, Tpo and Nis. Three to eight tissue samples were studied at each developmental stage. **(F)** Follicle size distribution among follicles >3000 μm² and thyroid surface area reported to animal weight at 4-months and 18 months-old (five to eight mice with each genotype). Note the increase in the number of large follicles and thyroid surface area at 18-months compared to 4-months in WT and *Borealin* ^+/-^ groups. Note the significantly greater size heterogeneity in the *Borealin* ^+/-^ group at 18-months. **(G)** List of primers sequence used for quantitative real-time PCR. **(H)** Expression of *BOREALIN/CDCA8* according to the thyroid differentiation score. Data retrieved from TCGA (9). Note that the expression of *BOREALIN* increased when the score is higher.
