# Supplementary material for: Borealin/CDCA8 deficiency alters thyroid development and results in papillary tumor-like structures
Source: Front Endocrinol (Lausanne). 2023 Oct 27;14:1286747. doi: 10.3389/fendo.2023.1286747 (PMC10641986; doi:10.3389/fendo.2023.1286747)

SUPPLEMENTARY MATERIAL

a

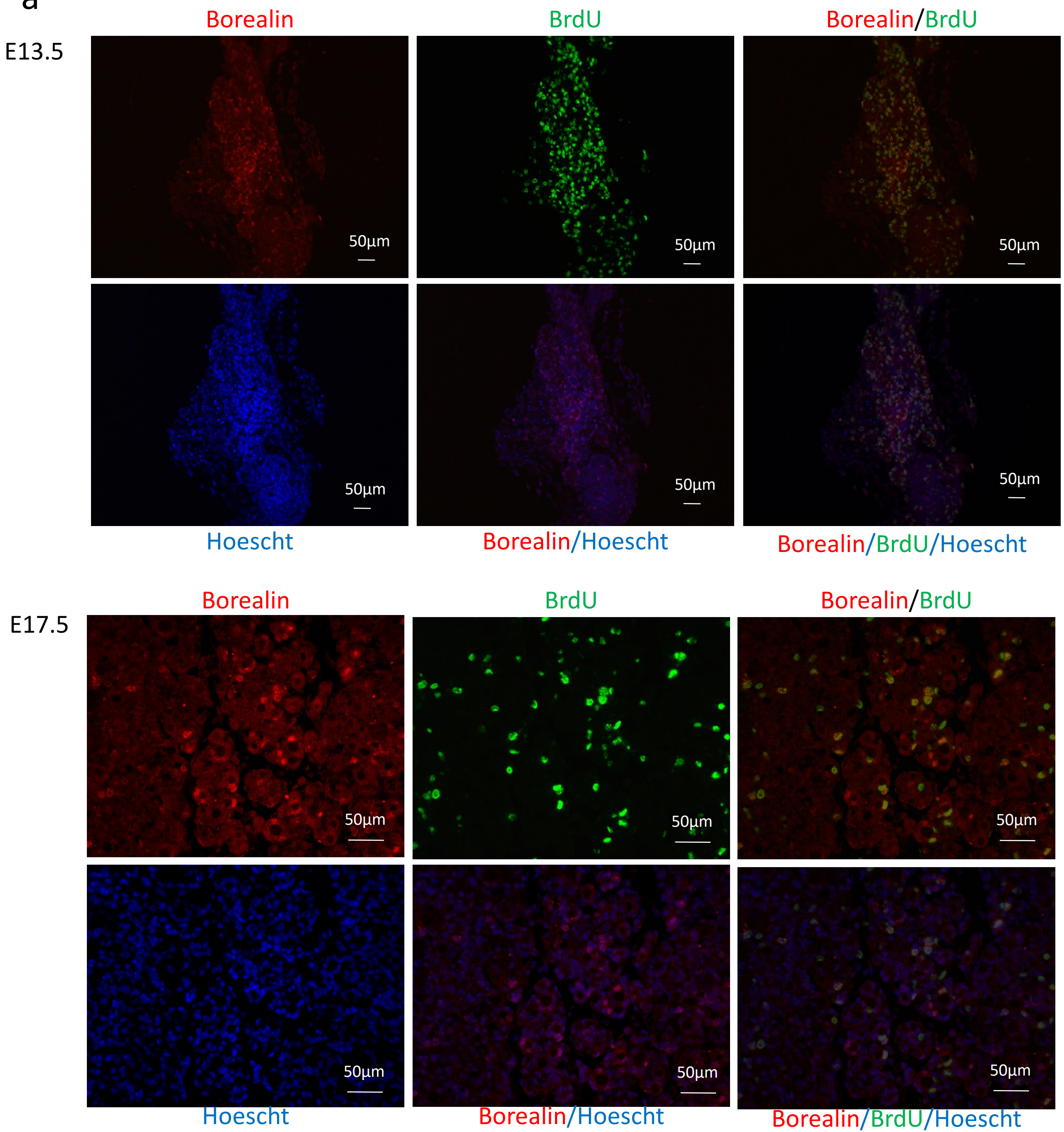

SUPPLEMENTARY MATERIAL

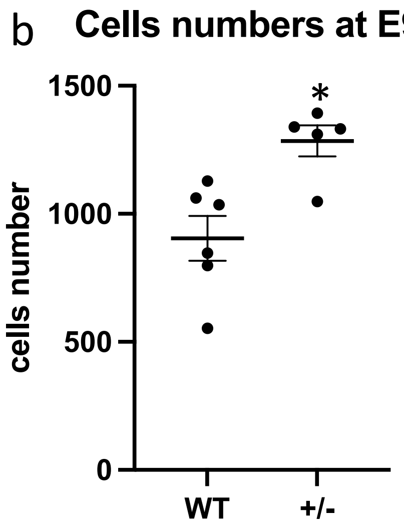

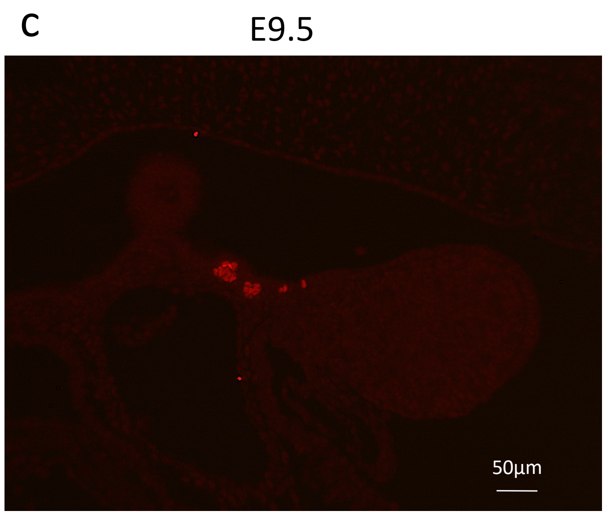

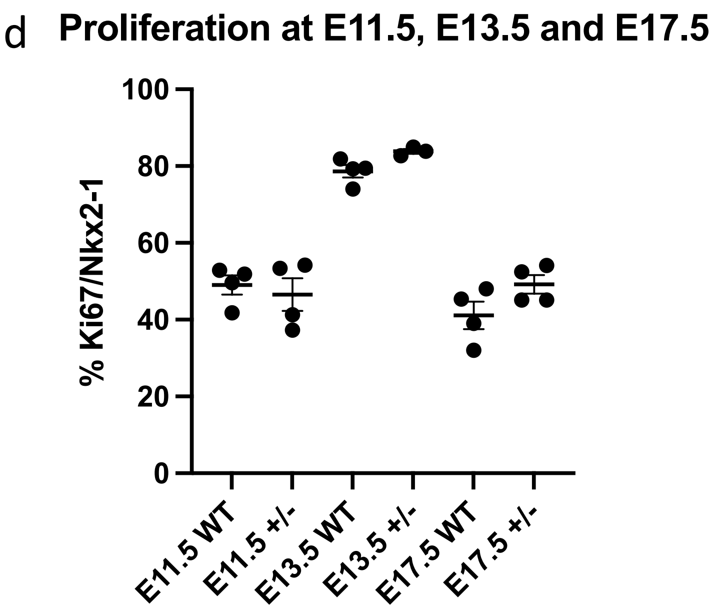

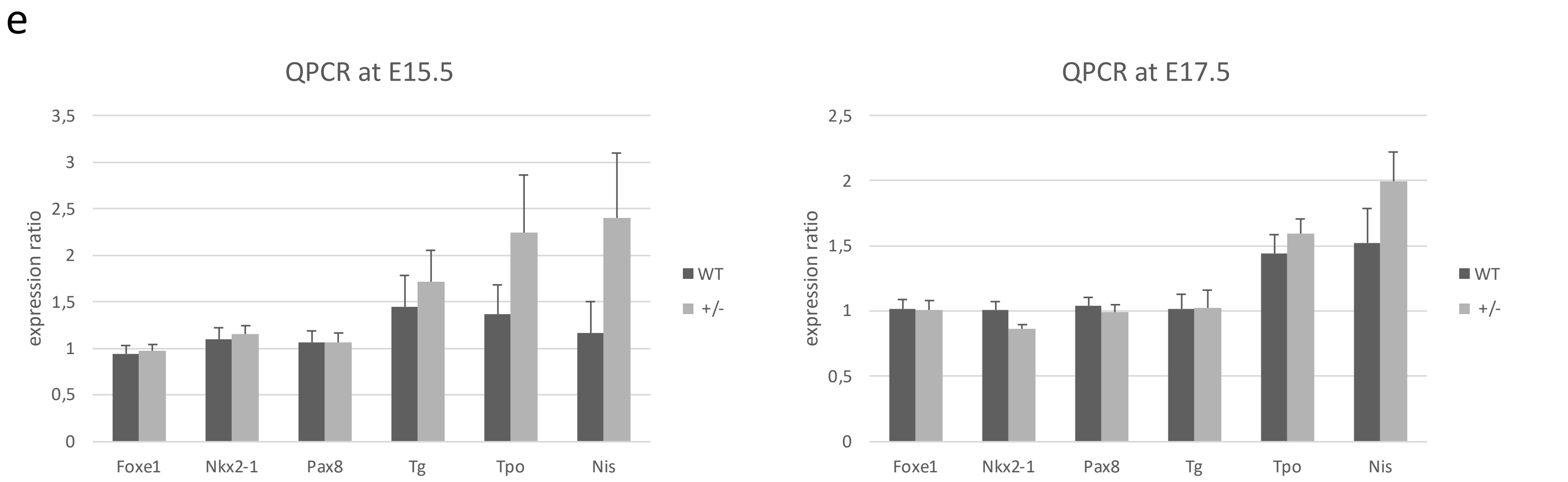

SUPPLEMENTARY MATERIAL

f. Size distribution of follicles >3000 µm² and thyroid surface area in mice aged 4 and 18-months

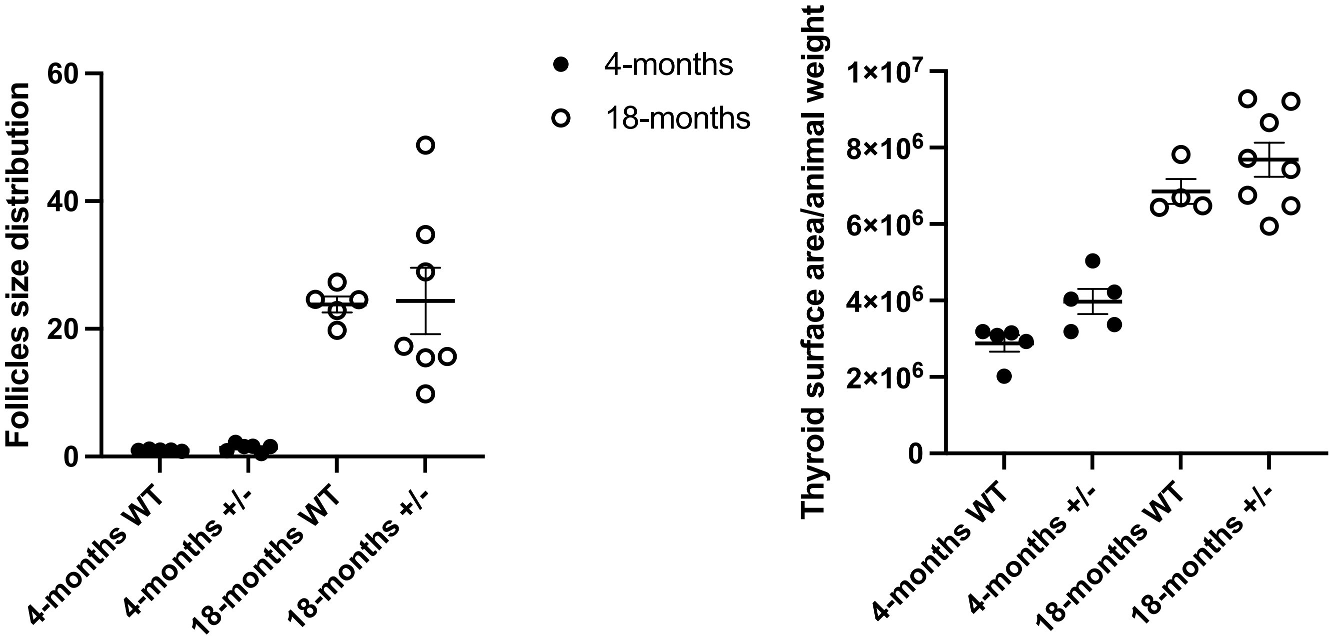

g. List of primers

	F	R
Borealin	TGTGGAGTGACTGAGAACCGTCTA	ACAGACCCTACTGCTGACTGCATA
Foxe1	CTACAAGTTCATCACCGAGC	GATCTTGAGGAAGCAGTCGT
Nkx2-1	GTTCAGACTCAGTTCCACTC	ACTTTCTTGTAGCTTTCCTCCA
Pax8	CCGGCGATGCCTCACA	AAAGGCCCCTCCTAGTTGATTC
Tg	TCAGGAAGGCACTGCTTATGG	GCCCTCTCTGGGCTGATAATT
Tpo	ACAAGGCCGCAGCTCAAG	CAATGTCTGGCTCCAAAGCA
Nis	TCTACTACAAGGACTGCGAC	CCATAGATGAATGAGAGCCC

SUPPLEMENTARY MATERIAL

h. Retrieved data from TCGA, 2014

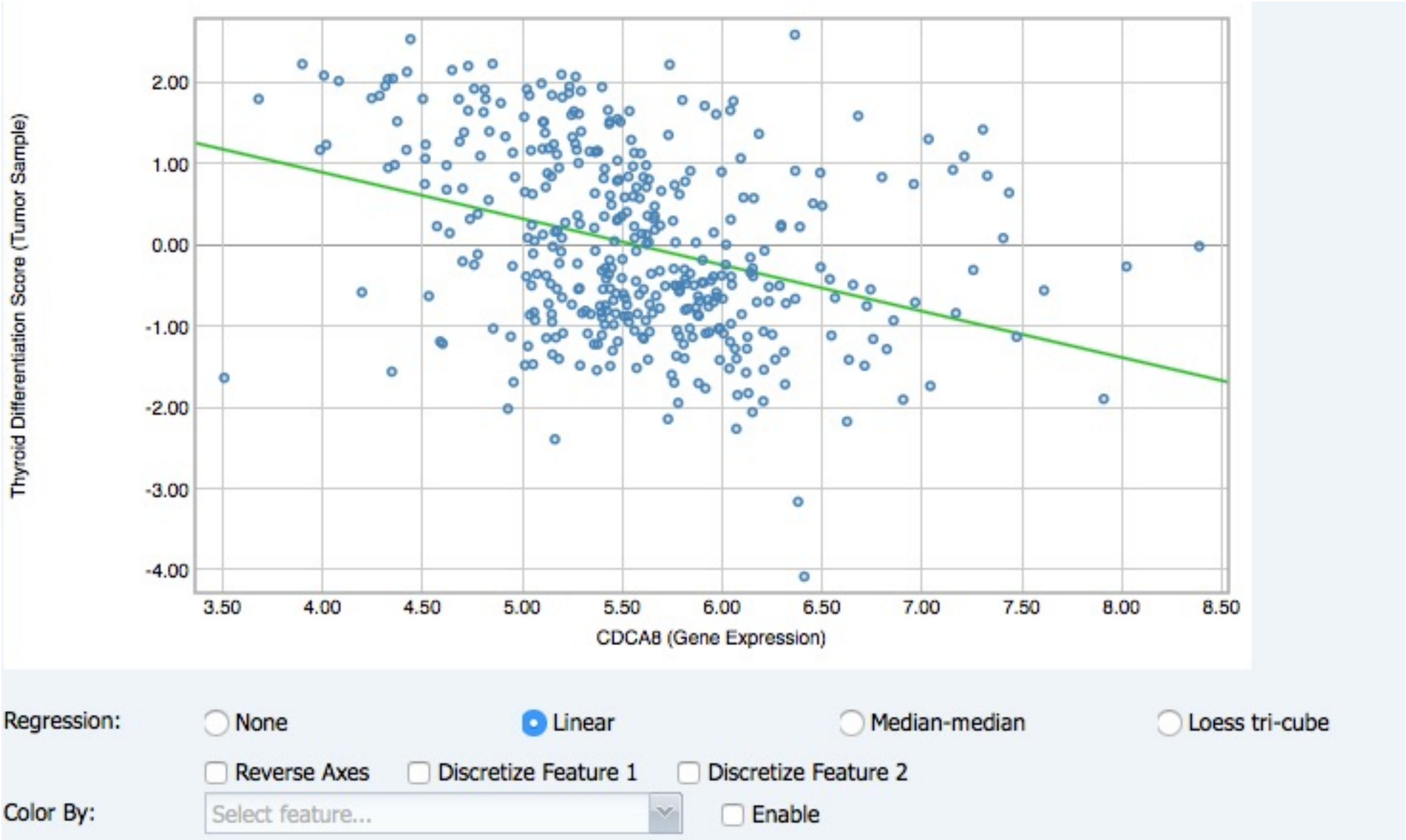

Supplement: Supplementary Figure — (A) Immunohistofluorescence staining for Borealin (in red) and BrdU (in green) in thyroid tissue on E13.5 and E17.5. Hoechst stained nuclei in blue. Note the Borealin expression in nuclei of thyrocytes progenitors and thyrocytes (co-staining Borealin/Hoescht/BrdU). (B) Number of cells Nkx2-1-positive in median anlage at E9.5. Note the increase of number of progenitors in Borealin +/ - compared to WT. P<0.05. (C) Thyroid morphology using Nkx2-1 staining on E9.5 (sagittal sections) of Borealin +/- littermate. Note thyroid anlage fragmentation at E9.5 in this Borealin +/- embryo. to: tongue. (D). Proliferation ratio calculated as the proportion of Nkx2-1-positive cells labelled with Ki67 on E11.5, E13.5 and E17.5: no significant difference. Three to four tissue samples were studied at each developmental stage. (E) Quantitative PCR assessment of thyroid markers expression by thyroid tissue on embryonic days E15.5 and E17.5 normalized for peptidylpropyl isomerase A and WT. Thyroid markers: Foxe1, Nkx2-1, Pax8, Tg, Tpo and Nis. Three to eight tissue samples were studied at each developmental stage. (F) Follicle size distribution among follicles >3000 μm² and thyroid surface area reported to animal weight at 4-months and 18 months-old (five to eight mice with each genotype). Note the increase in the number of large follicles and thyroid surface area at 18-months compared to 4-months in WT and Borealin +/- groups. Note the significantly greater size heterogeneity in the Borealin +/- group at 18-months. (G) List of primers sequence used for quantitative real-time PCR. (H) Expression of BOREALIN/CDCA8 according to the thyroid differentiation score. Data retrieved from TCGA (9). Note that the expression of BOREALIN increased when the score is higher. [file DataSheet_1.pdf]
